# Diabetic angiopathy and angiogenic defects

**DOI:** 10.1186/1755-1536-5-13

**Published:** 2012-08-01

**Authors:** Ling Xu, Keizo Kanasaki, Munehiro Kitada, Daisuke Koya

**Affiliations:** 1 Division of Diabetology & Endocrinology, Kanazawa Medical University, Uchinada, Ishikawa, 920-0293, Japan; 2 Division of Diabetology & Endocrinology, The Affiliated Hospital of Luzhou Medical College, Luzhou, Sichuan Province, 646000, Peoples’ Republic of China

**Keywords:** Diabetes, Complication, Angiogenesis, VEGF

## Abstract

Diabetes is one of the most serious health problems in the world. A major complication of diabetes is blood vessel disease, termed angiopathy, which is characterized by abnormal angiogenesis. In this review, we focus on angiogenesis abnormalities in diabetic complications and discuss its benefits and drawbacks as a therapeutic target for diabetic vascular complications. Additionally, we discuss glucose metabolism defects that are associated with abnormal angiogenesis in atypical diabetic complications such as cancer.

## Review

The epidemic of obesity-associated type 2 diabetes has prompted the need for strategies to prevent and treat diabetic complications [1]. In diabetes, diverse sets of organs are damaged. Such organ damage is certainly fundamentally associated with glucose metabolism defects. Therefore, normalizing blood glucose levels is essential for diabetic therapy [2-4]. However, recent evidence suggests that normalization of blood glucose levels is challenging in diabetes, and such intensive therapies in diabetic patients are associated with increased mortality risk, likely associated with frequent hypoglycemia [5]. To this end, patients enrolled in the intensive therapy group of the ACCORD trial, which employed intensive blood glucose lowering strategies aimed to normalize blood sugar levels, exhibited increased mortality [5]. Therefore, to prevent diabetic complications, additional therapeutic strategies are required in addition to those that target blood glucose normalization.

Angiopathy is a term for vascular defects that are associated with angiogenic abnormalities [6]. Understanding the precise molecular mechanisms that lead to diabetic angiopathy is essential for designing new therapeutic strategies to treat diabetic complications. In this review, we focus on diabetic vascular defects and abnormal angiogenesis.

### Angiogenesis

**Figure 1:**
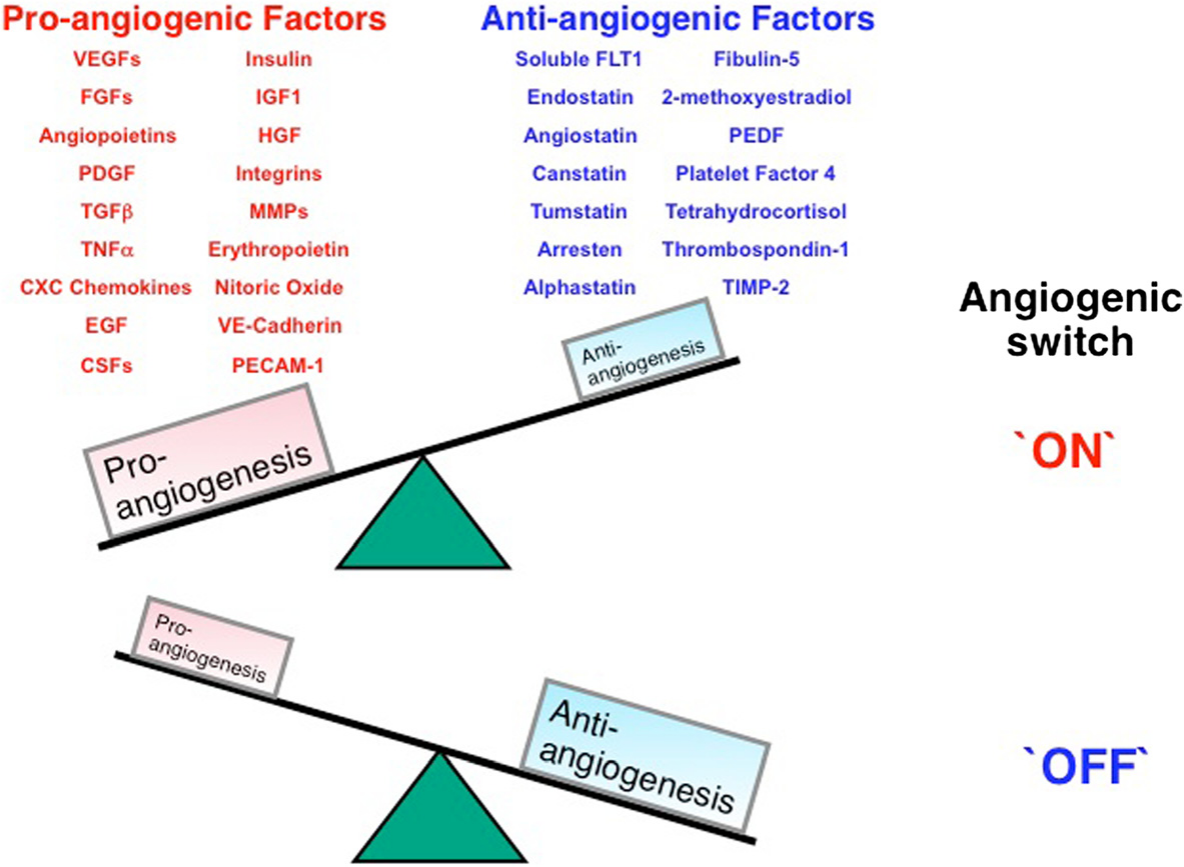
**Schematic image of angiogenesis switch.** Angiogenesis results from the balanced functions of pro-angiogenic and anti-angiogenic molecules. Defects in the angiogenic balance lead to a shift toward either excessive angiogenesis or anti-angiogenesis. CSF, colony-stimulating factor; EGF, epidermal growth factor; FGF, fibroblast growth factor; FLT1, fms-related tyrosine kinase 1; HGF, hepatocyte growth factor; IGF, insulin-like growth factor; MMP, matrixmetalloproteinases, PDGF, platelet-derived growth factor; PECAM-1, platelet endothelial cell adhesion molecular (also known as CD31); PEDF, pigment epithelium-derived factor; TGFβ, transforming growth factor-β; TIMP, tissue inhibitor of metalloproteinases; TNFa, tumor necrosis factor-α; VE, vascular endothelial; VEGF, vascular endothelial growth factor.

Angiogenesis is characterized by new blood vessel formation from pre-existing vessels and is distinguished from vasculogenesis, which is *de novo* vessel formation from hematopoietic progenitor cells [7]. Angiogenesis is essential for proper development and organ homeostasis, such as placental and embryonic growth, collateral formation, wound healing, and granulation [8]. However, angiogenesis is not always healthy and is often associated with pathologic conditions, in which case it is referred to as pathologic angiogenesis [7]. Angiogenesis results from the balanced functions of pro- and anti-angiogenic molecules (Figure 1). Defects in the angiogenic balance may cause a shift towards either excessive or anti-angiogenesis. Among angiogenic regulators, vascular endothelial growth factor (VEGF) has been associated with several diabetic complications, particularly diabetic retinopathy.

In diabetes, angiogenesis is regulated in an organ-, tissue-, and cell type-specific manner [9]. For example, in the retina, VEGF likely plays pro-angiogenic roles; thus, neutralizing VEGF is one anti-angiogenesis therapeutic strategy that is currently employed in clinical settings [10,11]. However, in the diabetic heart, VEGF signals are disturbed and collateral vessel formation is disrupted in spite of VEGF levels that are similar to those in non-diabetic subjects [12]. In cancer cells, high glucose induces the accumulation of hypoxia inducible factor (HIF)-1α and the associated expression of VEGF; however, in normal cells, such exposure to high glucose inhibits HIF-1α and VEGF expression [13,14].

### Abnormal angiogenesis and diabetic retinopathy

The abnormal angiogenesis that occurs in diabetic retinopathy has been well characterized. In diabetic retinopathy, the pericytes of the retinal capillaries are injured, which is associated with defective capillary function [15-19]. Such capillary deficiency is associated with defects in proper oxygen delivery and nutrient supply, resulting in VEGF overproduction in the retina [17]. This VEGF overproduction is also associated with abnormal angiogenesis and enhanced retinal capillary permeability, resulting in retinal dysfunction associated with the loss of visual acuity in these patients [17-19]. One therapeutic approach for diabetic retinopathy, light coagulation, has been performed for several years in clinical settings; however, this treatment is insufficient by itself.

**Figure 2:**
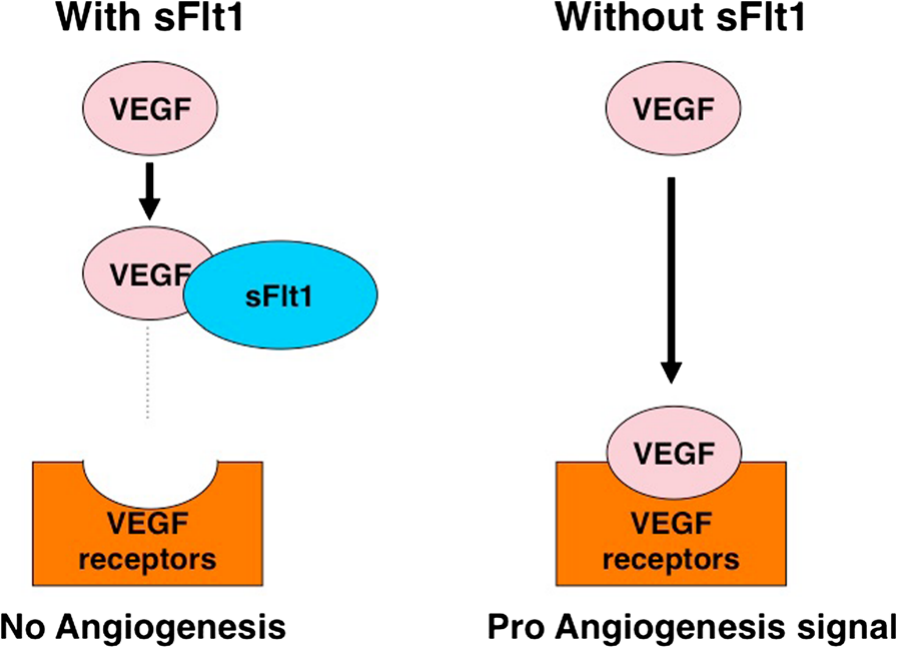
**sFllt1 plays as endogenous inhibitor of VEGF signaling by trapping free-VEGF.** VEGF signaling is strictly regulated by endogenous molecules, including sFlt1. sFlt1 binds to and sequesters VEGF from cell-surface VEGF receptors, subsequently VEGF modulated pro-angiogenesis signal is inhibited.

In the ocular system, VEGF signaling is strictly regulated. For example, the cornea is an avascular organ, and this lack of vascularity is regulated by abundant soluble VEGF receptor 1 (also known as sFlt1), which is a secreted protein that binds and sequesters VEGF from the VEGF receptors on the cell surface (Figure 2) [20]. The only mammal with a vascularized cornea is the manatee, which is due to a lack of corneal sFlt1 [20]. These properties of VEGF have enabled scientists to design molecules that target and normalize VEGF signaling using similar mechanisms to sFlt1, the endogenous VEGF blocker (Figure 2). Therefore, anti-VEGF molecules, such as pegaptanib sodium (Macugen), ranibizumab (Lucentis), and bevacizumab (Avastin) have been developed.

In 2004, the U.S. Food and Drug Administration (FDA) approved pegaptanib sodium for the treatment of age-related macular degeneration, in which abnormal VEGF signals are associated with abnormal angiogenesis and edema in the retina, similar to diabetic retinopathy [21]. Pegaptanib sodium was the first anti-VEGF drug approved for ocular disease. This provided seminal evidence that VEGF is responsible for the ocular diseases that are associated with abnormal angiogenesis [22]. In later clinical trials, ranibizumab was shown to be effective in more than 90% of the cases of age-related macular degeneration [23,24]. The FDA has approved bevacizumab to treat colorectal cancer, but it has not been approved for the treatment of ocular diseases; however, this drug is being tested clinically for treating age-related macular degeneration [25]. Anti-VEGF therapy is also effective for diabetic retinopathy [10], indicating a critical role for VEGF in the pathogenesis of this disease.

### Abnormal angiogenesis and diabetic nephropathy

Diabetic nephropathy is the leading cause of end-stage renal disease worldwide. The number of patients requiring hemodialysis because of diabetic kidney-associated diseases has increased tremendously over the past two decades. Once renal function has deteriorated, many associated cardiovascular events can occur [26-28]. Therefore, more research is needed to discover novel strategies to prevent or slow this decline in renal function. Furthermore, the therapeutic targeting of angiogenic abnormalities provides substantial clinical benefits. However, the contribution of VEGF to diabetic nephropathy-induced angiogenesis is complicated.

First, we review VEGF biology in the normal kidney, which is based on the experiences of cancer patients that have been treated with bevacizumab [29]. These patients displayed hypertension, edema, proteinuria, and glomerular capillary damage [30-32]. Similar renal microcirculation effects have been reported in rodents after human sFlt1 injection [33], adenovirus-mediated humans Flt1 overexpression [34], or endothelial specific VEGF deletion, which induced endothelial damage with microthrombi [35]. It is also hypothesized that the actions of anti-VEGF molecules might be associated with microcirculation injuries that occur in pre-eclampsia patients [34,36,37]. Therefore, VEGF is essential for the homeostatic maintenance of renal hemodynamics. In contrast, VEGF overproduction in the glomerular podocytes is associated with glomerular capillary collapse and HIV-associated glomerulopathy [38,39].

In diabetic nephropathy, abnormal angiogenesis in the glomeruli, as well as VEGF overexpression, has been reported, similar to diabetic retinopathy [40,41]. The properties of these abnormal vessels and how these vessels are associated with the pathogenesis of diabetic nephropathy are not well described. In experimental animal models, anti-VEGF therapy [41-44] or administering anti-angiogenesis molecules [45-49] may reverse such abnormal angiogenesis in diabetic kidneys, concomitantly reducing albumin excretion into the urine. However, recent evidence suggests that the neutralization of VEGF by sFlt1 in diabetic animal models ameliorates abnormal vasculature in the glomeruli, but in the interstitium, sFlt1-mediated VEGF neutralization caused the deterioration of pathological lesions [50]. These data demonstrate the complexity of the VEGF overproduction-associated angiogenic pathways in diabetic kidneys. Therefore, angiogenesis abnormalities in diabetic nephropathy progression are still controversial, and further research needs to be conducted to determine whether, and how, abnormal angiogenesis can be therapeutically targeted.

### Abnormal angiogenesis and atherosclerosis

Atherosclerosis-associated coronary artery disease is a major cause of mortality in diabetic patients. It is likely that plaques, the core atherosclerotic lesions, play essential roles in the onset of life-threatening coronary artery disease. The plaque begins as a fatty streak, an ill-defined yellow lesion-fatty plaque, which develops well-demarcated edges, and evolves to fibrous plaques, which are whitish lesions with a grumous lipid-rich core [51]. The rupture of these plaques following the enlargement of the necrotic core is associated with luminal thrombosis in acute coronary syndrome, which occurs in 75% of patients who die of an acute myocardial infarction [52]. However, the mechanisms by which asymptomatic fibroatheromatous plaques progress to high-risk, unstable lesions are not clear.

Intra-plaque hemorrhage may play an important role in the process of plaque destabilization [53]. Red blood cell (RBC) membranes are rich in phospholipids and free cholesterol, and RBC accumulation within the plaques plays an important role in the progression of plaque instability [54]. The RBC source within the coronary lesions is therefore important, and it is likely that leaky, immature vessels within the plaque allow the entry of RBCs into the lesions [54]. In the unstable or ruptured plaque, newly formed vessels are found in abundance [54]. Pathologic examination of unstable lesions has demonstrated that intraplaque hemorrhage and plaque rupture are associated with increased microvessel density. Although most intraplaque vasa vasorum are endothelialized, only a few have mural cells such as pericytes and vascular smooth muscle cells [55,56]. This lack of mural cells may contribute to vessel leakiness because such vessels are fragile and are therefore easily damaged. This damage results in the development of immature vessels within the lesion, which are associated with abnormal angiogenesis [57].

During plaque progression, the plaque becomes complicated and is composed of infiltrated inflammatory cells, smooth muscle cells, and extracellular matrix in the large artery intima [57]. Inflammatory cells such as T-cells and macrophages may contribute to VEGF production within the lesion [57]. Plaque progression may be associated with decreased oxygen and nutrient supply within the lesion [58-60], which directly leads to HIF-1α accumulation and the subsequent induction of pro-angiogenic molecules. Therefore, inflammation and hypoxia within plaque lesions could activate angiogenesis and contribute to the plaque instability that is associated with abnormal angiogenesis.

### Wound healing defects in diabetes and angiogenesis

Wound healing capacity in diabetic patients is decreased. The normal process of wound healing is characterized by five sequential processes: (1) hemostasis, (2) inflammation and debridement, (3) proliferation, (4) epithelialization, and (5) remodeling [61]. The delayed wound healing in diabetic patients has been attributed to disturbances in the inflammation/debridement and proliferation phases [61,62]. In the wound healing process, microangiopathy may also contribute to defects in the nutrient/oxygen supply, thus inhibiting normal healing processes [63]. Both clinical research and animal models have confirmed such wound healing defects in diabetes.

In diabetic patients, chronic non-healing ulcers are frequently observed at pressure points of the lower extremities [62]. Pathological analysis has revealed abnormal microvessels that can be cuffed with collagen, laminin, fibronectin, or fibrin in the wound edges of these diabetic ulcers [64]. Fibroblasts isolated from diabetic ulcers display diminished proliferative capacity and abnormal morphological features, such as multiple lamellar and vesicular bodies, an absence of microtubular structures, and enlarged, dilated endoplasmic reticulum, indicative of a hypertrophic phenotype [61]. Such alterations would be functionally relevant to angiogenic defects within the wound because fibroblasts play essential angiogenic roles by producing several pro-angiogenic cytokines such as VEGF and fibroblast growth factors [65], and because microtubules are important for fibroblast migration [66,67].

Another possible cause of wound healing defects in diabetes patients has been associated with altered biology of bone marrow-derived endothelial progenitor cells (EPCs) [68,69]. EPCs are thought to be essential in vasculogenesis and wound healing, but their functions and numbers in the circulation and within wounds have been shown to be compromised in diabetic patients [68-75]. Defects in the recruitment of EPCs for re-endothelialization has been suggested in diabetes patients [76]. VEGF signaling, matrix metalloproteinases, and endothelial nitric oxide synthase (eNOS) have been shown to play essential roles for the recruitment of EPCs into wounds [77,78]. Recently, Albiero *et al.*. showed that EPCs in diabetic patients exhibited both proliferative defects and enhanced apoptosis without altering the number of circulating EPCs [79], suggesting that diabetes affects EPC survival signaling, and such a survival defect could be a potential therapeutic target for treating defects in diabetic wound healing.

### Abnormal angiogenesis and cancer

Diabetes increases mortality risk in cancer patients [80,81]. Cancer patients who already have diabetes reportedly have a greater chance of dying of the cancer than those who do not [80]. Furthermore, cancer patients with preexisting diabetes exhibit approximately a 50% greater risk of dying after surgery [81]. There are many theories for this diabetes-associated increase in mortality, such as possible links to glucose-mediated cancer growth, immunodeficiency, infections, or other health problems. Diabetes is also associated with the diagnosis of more advanced cancers [82]. Therefore, there are possible links between cancer-accelerating factors and diabetes.

Several clinical trials have demonstrated that anti-angiogenesis therapy is beneficial for cancer treatment [83], suggesting that increased angiogenic signals contribute to cancer progression. Tumor hypoxia is a strong angiogenesis inducer via accumulation of HIFs and their downstream targets, such as VEGF. These angiogenic abnormalities may be relevant to the association between cancer and diabetes. In tumor cells, high levels of glucose induce the accumulation and expression of HIF-1α, whereas non-tumor cells exhibit decreased HIF-1α accumulation in response to high glucose [13,14], suggesting that impaired glucose homeostasis directly affects angiogenic signals within tumors.

Type 2 diabetes is characterized by insulin resistance and hyperinsulinemia. Hyperinsulinemia induces breast cancer development in experimental animal models [84]. Type 2 diabetes is often associated with obesity, which is another risk factor for cancer [85]. Additionally, patients with type 2 diabetes exhibit increased levels of insulin-like growth factor (IGF)-1, a potent mitogen and pro-angiogenic factor that may contribute to carcinogenesis [86]. IGF-1 promotes liver metastasis in xenograft colon adenocarcinoma models in obese mice [87]. Furthermore, insulin resistance in type 2 diabetes is associated with diacylglycerol (DAG) accumulation in cells [88,89]. DAG accumulation can cause activation of the protein kinase C family of serine-threonine kinases [89], which play important roles in cancer biology and abnormal angiogenesis in diabetic patients [90].

In cancer biology, angiogenesis is closely connected with inflammation [91,92]. Recently, Park *et al*. reported that enhanced inflammation in obesity is associated with liver carcinogenesis [93]. They used leptin-deficient *ob/ob* mice and high-fat diet (59% fat, 15% protein, 26% carbohydrate)-induced obesity models and found that diethylnitrosamine-induced hepatocellular carcinoma (HCC) is significantly advanced in the both of these murine obesity models [93]. The high-fat diet also resulted in increased growth of subcutaneous-injected HCC [93]. The mechanisms of obesity-induced liver carcinogenesis were found to be associated with hepatic activation of the Stat signaling pathway and inflammation [93]. To this end, depleting inflammatory cytokines interleukin-6 or tumor necrosis factor-α in the liver of obese mice abolished the tumor-promoting effect of obesity, thus suggesting a critical role of inflammation in obesity-associated carcinogenesis [93].

Leptin levels are often elevated in obesity-induced type 2 diabetes, which could be another possible connection to abnormal angiogenesis and cancer. Leptin induced endothelial cell proliferation both *in vivo* and *in vitro*[94]. Using androgen-insensitive murine prostate carcinoma RM1 cells, Ribeiro *et al*. recently reported that *ob/ob* mice, which lack leptin, and diet-induced obese mice exhibited large tumors. Conversely, *db/db* mice, which express leptin but have a mutation in the leptin receptor, displayed small tumors, suggesting that leptin has a tumor-suppressive role [95]. However, Gonzalez *et al*. reported that leptin may accelerate murine breast tumor growth because it induces VEGF-mediated angiogenesis [96], even though the mouse model used in this study was the immunodeficient SCID mouse, which is neither diabetic nor obese [97]. Similarly, leptin induced proliferation and invasiveness of endometrial cancer cells [98]. Recently, Bartucci *et al*. found that the leptin receptor is expressed on colorectal cancer stem cells; therefore, leptin may induce tumor growth and interferes with the cytotoxic effects of the anti-cancer drug 5-FU [99].

### Diabetes in pregnancy and vascular defects of the embryo and placenta

The vasculature is the first embryonic system to develop and is vulnerable to insults from the uterine environment. Hyperglycemia is associated with embryonic vasculopathy, which may lead to embryonic lethality or malformation [100-106]. The molecular mechanisms underlying maternal diabetes-induced embryonic vasculopathy are unclear. Several *in-vitro* and *ex-vivo* studies suggest that exposure to high glucose induces anomalies in the yolk sac microvasculature [107,108]. Embryos in streptozotocin-injected pregnant female mice exhibit abnormal angiogenesis and vasculogenesis [108]. Recently, Yang *et al*. reported that decreased accumulation of HIF-1α in the conceptus when cultured in high-glucose media might be associated with decreased VEGF, thus suggesting that HIF-1α homeostasis may be the key to understanding embryonic diabetes-induced vasculopathy [109].

Developing the placental vasculature is essential for the developmental homeostasis of the placenta and fetus. Defects in the placental vasculature are associated with placental hypoxia, which may result in the onset of pre-eclampsia [110], the devastating pregnancy-associated hypertensive syndrome. Maternal hyperglycemia, caused by either pre-existing or pregnancy-induced diabetes, has been associated with increased incidence of placental defects and pre-eclampsia. Morphologically, diabetic pregnancy is characterized by reduced fetal capillary branching, maldevelopment of the villous tree, and impaired adaptation of maternal vasculature during pregnancy [111-117]. Diabetic mothers without pre-eclampsia tend to have decreased levels of pro-angiogenic molecules [118]. However, the poor development of the placental vasculature in pre-eclampsia is most likely not a result of imbalance among VEGF signaling pathways, but rather of complex interactions among maternal spiral arteries and the trophoblast [110,119-121]. Inflammation in the maternal-fetal interface is also essential for development of the placental vasculature [122-125]. Therefore, all of these molecules may be relevant in the placental and embryonic angiogenesis defects that occur in diabetic pregnancies. These points should be clarified by further research.

### Perspective

In this review, we summarized and discussed diabetic angiopathy while focusing on angiogenic defects. Systemic angiogenesis modification therapies that either inhibit or activate angiogenesis are not acceptable therapeutic strategies because of the potential adverse reactions that may occur. Therefore, there is a need to target locally acting molecules, such as VEGF, to treat diabetic retinopathy. To this end, inhibition of ocular VEGF has emerged as a promising treatment modality for diabetic retinopathy and is currently being evaluated in clinical trials. However, anti-VEGF therapy for the treatment of diabetic retinopathy is of limited use and involves potential adverse reactions such as retinal ischemia, vasoconstriction, inflammation or detachment [126-131]. Another possible strategy for treating VEGF-mediated angiogenesis defects could be to target mediators of VEGF intracellular signaling pathways such as phosphoinositide 3-kinase, Akt, protein-kinase C, mitogen activated protein kinases, or nitric oxide. However, avoiding potential adverse effects would be essential and tissue specificity could be an important issue.

We focused on the role of the VEGF system in diabetic angiopathy and angiogenetic defects in this review. However, other molecules contribute to abnormal angiogenesis in diabetes. As shown in Figure 1, various pro- and anti-angiogenic molecules could be relevant in the pathogenesis of diabetes-induced angiogenesis defects. The role of fibroblast growth factors and angiopoietins in the onset of diabetic nephropathy and/or retinopathy has been previously demonstrated [132]. A potential for targeting several other endogenous anti-angiogenic factors such as platelet factor-4, angiostatin, endostatin, vasostatin, and tumstatin has also been described for preclinical diabetic angiopathy treatment [132]. These are all potent, significant molecules, and further research is required to determine how these findings can be applied in clinical settings.

## Conclusion

**Figure 3:**
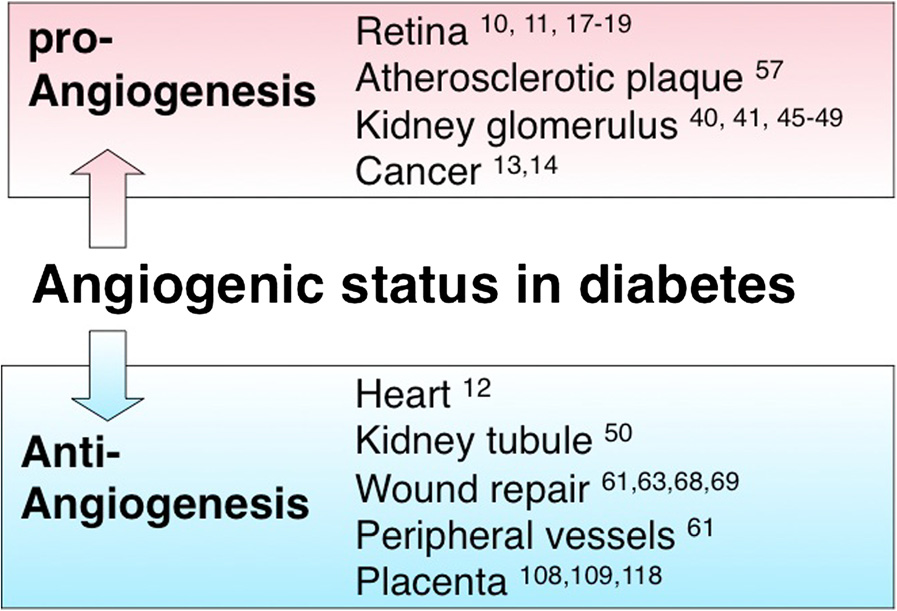
**The biology of angiogenesis abnormality in diabetic organ dysfunction.** In diabetes, the angiogenesis signal is regulated in an organ-, tissue-, and cell type-specific manner. In the retina, atherosclerotic plaque, kidney glomerulus, and cancer, VEGF likely plays pro-angiogenic roles; on the contrary, in diabetic heart, kidney tubule, peripheral vessels, and placenta, VEGF signal is inhibited.

In diabetes, the VEGF response likely depends on the cell type and organ (Figure 3). Additionally, hypoxic responses and the induction of the master hypoxia transcription factor, HIF-1α, depends on the cell type [13,14]. Altered angiogenesis is a well-defined pathogenesis of diabetic angiopathy, although to therapeutically target angiogenesis defects, further research to identify tissue, organ, and disease-specific molecules is necessary.

## Abbreviations

ACCORD, Action to Control Cardiovascular Risk in Diabetes; eNOS, Endothelial Nitric Oxide Synthase; EPC, Endothelial progenitor cells; HIF-1α, Hypoxia inducible factor-1α; IGF, Like growth factor; sFlt1, Soluble fms-like tyrosine kinase-1 (same as VEGF type1 receptor); VEGF, Vascular endothelial growth factor.

## Competing interests

The authors declare that they have no competing interests.

## Authors’ contribution

LX contributed to writing the manuscript and made figures. MK was involved in the discussion. DK made intellectual contributions. KK conceived the project, provided intellectual contribution, and contributed to the manuscript writing and editing. All authors read and approved the final manuscript.
